# Mass Spectrometry-Based Metabolomic and Lipidomic Analysis of the Effect of High Fat/High Sugar Diet and Greenshell^TM^ Mussel Feeding on Plasma of Ovariectomized Rats

**DOI:** 10.3390/metabo11110754

**Published:** 2021-10-31

**Authors:** Maryam Abshirini, Diana Cabrera, Karl Fraser, Parkpoom Siriarchavatana, Frances M. Wolber, Matthew R. Miller, Hong Sabrina Tian, Marlena C. Kruger

**Affiliations:** 1 School of Health Sciences, College of Health, Massey University, Palmerston North 4442, New Zealand; Maryam.Abshirini.1@uni.massey.ac.nz; 2 Food Chemistry and Structure, AgResearch Grasslands, Palmerston North 4442, New Zealand; Diana.Cabrera@agresearch.co.nz (D.C.); karl.fraser@agresearch.co.nz (K.F.); 3 High-Value Nutrition National Science Challenge, Auckland 1023, New Zealand; 4 Riddet Institute, Massey University, Palmerston North 4442, New Zealand; 5 School of Food and Advanced Technology, Massey University, Palmerston North 4442, New Zealand; blueno00@gmail.com (P.S.); F.M.Wolber@massey.ac.nz (F.M.W.); 6 Centre for Metabolic Health Research, Massey University, Palmerston North 4442, New Zealand; 7 Cawthron Institute, Nelson 7010, New Zealand; matt.miller@cawthron.org.nz; 8 Sanford Ltd., Auckland 1010, New Zealand; STian@sanford.co.nz

**Keywords:** osteoarthritis, metabolic dysregulation, diet-induced obese rats, metabolomics, lipidomic

## Abstract

This study aimed to examine the changes in lipid and metabolite profiles of ovariectomized (OVX) rats with diet-induced metabolic syndrome-associated osteoarthritis (MetOA) after supplementation with greenshell mussel (GSM) using an untargeted liquid chromatography-mass spectrometry (LC-MS) metabolomics approach. Ninety-six rats were fed with one of four diets: control, control supplemented with GSM + GSM, high fat/high sugar (HFHS), or high fat/high sugar enriched with GSM (HFHS + GSM). After 8 weeks on experimental diets, half of the rats in each group underwent OVX and the other half were sham operated. After being fed for an additional 28 weeks, blood samples were collected for the metabolomics analysis. Lipid and polar metabolites were extracted from plasma and analysed by LC-MS. We identified 29 lipid species from four lipid subclasses (phosphatidylcholine, lysophosphatidylcholine, diacylglycerol, and triacylglycerol) and a set of eight metabolites involved in amino acid metabolism (serine, threonine, lysine, valine, histidine, pipecolic acid, 3-methylcytidine, and cholic acid) as potential biomarkers for the effect of HFHS diet and GSM supplementation. GSM incorporation more specifically in the control diet generated significant alterations in the levels of several lipids and metabolites. Further studies are required to validate these findings that identify potential biomarkers to follow OA progression and to monitor the impact of GSM supplementation.

## 1. Introduction

Osteoarthritis (OA) has been updated from being defined as a mechanical “wear and tear” disease to a metabolic disorder [1]. Emerging evidence has shown the involvement of metabolic components in OA pathogenesis [2]. Metabolic syndrome, which is characterized by hypertension, dyslipidemia, and diabetes, has been closely associated with low-grade systemic inflammation. Obesity and metabolic syndrome lead to a particular phenotype of OA known as metabolic syndrome-associated osteoarthritis (MetOA), which results in the development of microstructural damage in the joints, articular cartilage deterioration, and synovial endothelium dysfunction [3,4].

In order to understand and explore the MetOA, various diet-induced obesity models have been developed and validated in vivo. A high-fat diet has been shown to accelerate the progression of OA in both spontaneous and surgically-induced OA models [5]. It has been demonstrated that feeding rats with a high fat, high sucrose (HFHS) diet for 12 weeks establishes OA-like cartilage alteration, with systemic and local synovial inflammation along with visceral fat accumulation [6]. Furthermore, sex is a crucial factor in OA development as males are more predisposed to OA than females owing to the effect of testosterone, while females are at higher risk following menopause owing to oestrogen deficiency [7]. A high energy diet and lack of estrogen can have additive or synergistic effects, as we have previously shown that the combination of both factors caused a more severe pathological lesion in knee cartilage of rats than each factor individually [8]. 

Metabolomics approaches allow the profiling of small molecular metabolites from several metabolic pathways, providing a powerful tool for early diagnosis, monitoring intervention efficacy, and understanding the pathogenesis of diseases. [9]. The application of metabolomics is increasingly used in OA studies to understand the metabolism of disease and to identify new biomarkers. Some biomarkers have been identified by previous studies; for example, a pre-clinical study using an obese mouse model of OA demonstrated that serum levels of n-3 polyunsaturated fatty acids (PUFAs) were inversely associated with OA, while most n-6 PUFAs were positively associated with OA. Moreover, serum and synovial fluid levels of pentadecylic acid (C15:0, an odd-chain SFA) and palmitoleic acid in this study were negatively correlated with joint degradation [10]. Another study identified that mice fed a high-fat diet to induce OA showed a distinct and sustained plasma metabolite signature high in phosphatidylcholines (PC) and lysophosphatidylcholines (lysoPCs) [5]. However, metabolic changes induced by HFHS diet combined with ovariectomy that contribute to OA are not well understood.

Green-lipped mussel (*Perna canaliculus*) or Greenshell^TM^ mussel (GSM) is a commercial marine species endemic to New Zealand (NZ) waters. GSM is a rich source of long-chain omega-3 PUFAs [11], which are proven to have potent anti-inflammatory and anti-arthritic properties [12]. Our previous study has shown that flash-dried powder from whole GSM could prevent OA induction or slow its progression in a rat model of MetOA. Briefly, inclusion of GSM in the diet of rats fed with HFHS for 13 weeks significantly reduced the c-terminal cross-linked telopeptide of type II collagen (CTX-II), a biomarker of cartilage degradation [13]. After 36 weeks, GSM supplementation significantly attenuated the pathological cartilage lesion scores in the knee joints of ovariectomized (OVX) rats [8]. Siriarchavatana et.al [13] also identified an increase in lean mass gain and decrease in fat mass gain in rats fed with HFHS when GSM was incorporated into the diet. In line with this, Vaidya et al. reported reduced body weight gain, a decrease in systemic inflammation, and improved metabolic parameters in rats fed with HFHS diet supplemented with freeze-dried powder of blue mussel for 12 weeks [14]. Although these recent studies have provided evidence for advantageous effects of GSM on body weight, fat deposition, and metabolism, it remains unclear what broader impact GSM has on metabolic regulation. In the present study, liquid chromatography coupled to mass spectrometry (LC-MS) untargeted metabolomics was applied to reveal the impact of HFHS diet and GSM feeding on lipid and metabolite profiles in rats subjected to ovariectomy or sham surgery, in order to gain a better understanding of the beneficial effect of GSM in HFHS-induced obesity as a model of MetOA.

## 2. Results

This is the first study to investigate the effects of including whole meat GSM in the diet on plasma lipid and polar metabolites. Female rats fed with a normal (control) or HFHS diet and subjected to OVX or a sham surgery were used to model MetOA. The lipid and metabolite profiles were obtained from 96 female rats divided into four diet groups and subjected to surgical procedures as described previously [8]. HFHS diet markedly changed the lipidomic and metabolomic profile of rats subjected to OVX or sham, while treatment with GSM regulated some of these changes and reverted them to the levels observed in control diet groups.

**Figure 1:**
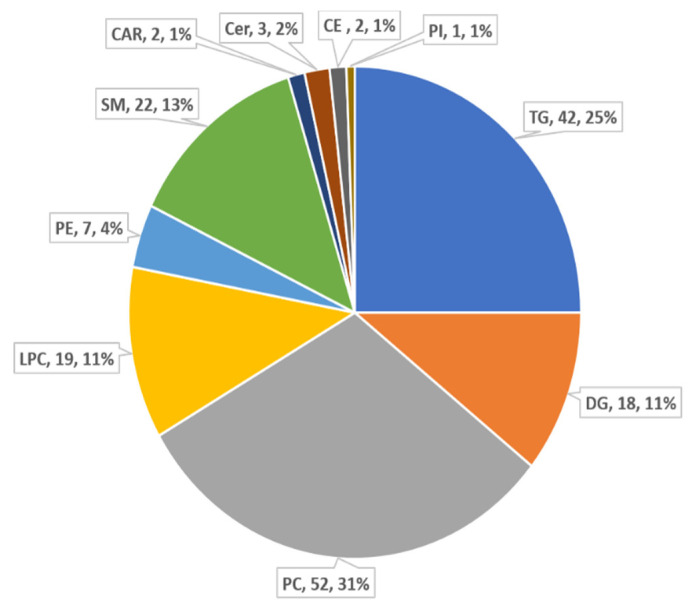
Composition of lipid classes that were considered for subsequent analysis in samples detected by LC-MS.

The lipidomic data were analysed in positive mode and a total of 721 features was detected. After removal of noise and unstable molecules, 168 different lipid species consisting of 52 phosphatidylcholines (PC) and 42 triglycerides (TG) and other lipid classes were identified (Figure 1). To obtain the global information regarding lipidomic change, diet groups were analysed for OVX and sham rats separately. PCA was performed to visualize the grouping trends and detect outliers (Figure S1). PCA plots of diet groups for OVX rats demonstrated a separation between HFHS and control diet groups, with PC1 explaining 50.2% of the variance, while PCA for sham rats did not show a defined distinction. In OVX rats, two outliers from HFHS and control + GSM diet and, in sham rats, three outliers (two from control diet and one from Control + GSM), were observed. These samples were retained for the analyses as they could represent part of natural inter-animal variation among the samples.

**Figure 2:**
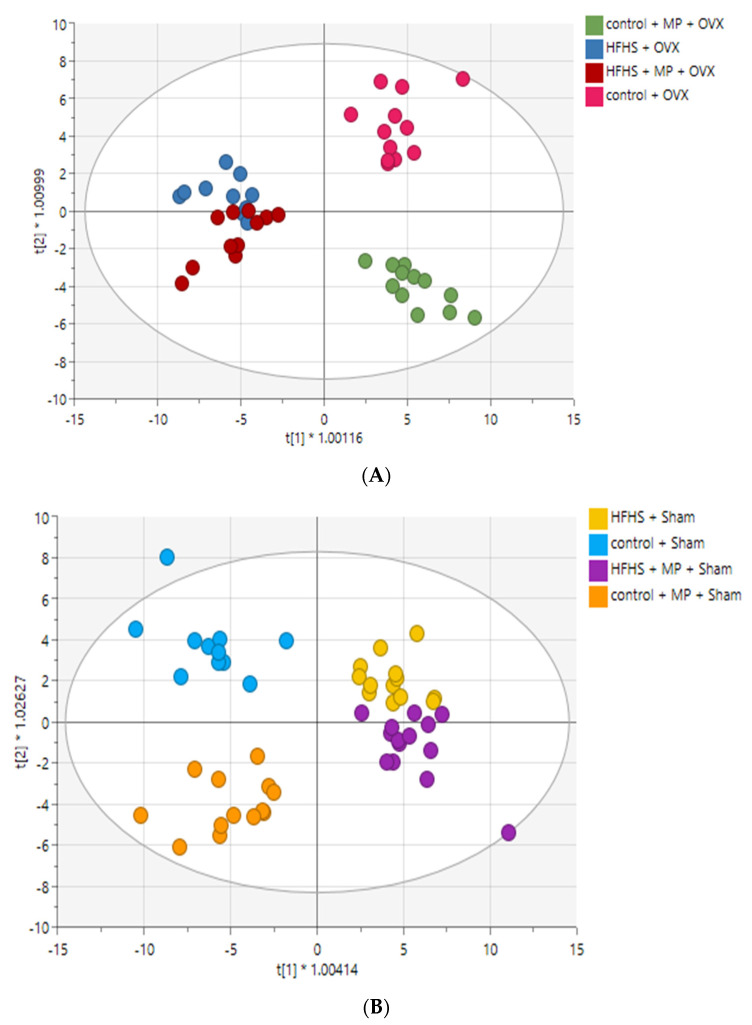
OPLS-DA analysis for lipidomic differences between diet groups showing good separation and robust modelling for sham rats and OVX rats. Each circle represents the lipid profile of a single rat. MP = greenshell mussel powder included in the diet. (**A**) OPLS-DA scoring plot showing discrimination between diets groups in OVX rats (*n* = 45); R2X (cum) = 0.811, R2Y (cum) = 0.608, Q2 (cum) = 0.498. Cross-validated ANOVA *p*-value = 4.38326 × 10^−5^. (**B**) OPLS-DA scoring plot showing discrimination between diets groups in sham rats (*n* = 51); R2X (cum) = 0.755, R2Y (cum) = 0.572, Q2 (cum) = 0.480. Cross-validated ANOVA *p*-value = 8.77889 × 10^−6^.

The results of the OPLS-DA revealed clear differences among the four treatment groups in OVX and in sham rats. The HFHS and control diet groups were discriminated from each other, while the HFHS + GSM diet was located near to or with minor overlap with the HFHS diet (Figure 2A,B). OPLS-DA models were used to select the discriminating variables between the four experimental diet groups. Those lipids that satisfied both VIP > 1.0 and FDR < 0.05 were further investigated. In OVX rats, 29 lipids were tentatively identified as biomarker candidates for HFHS-induced obesity or protective effects of whole meat GSM powder. 

Given the potential therapeutic value of GSM for MetOA, GSM supplementation may have regulated the lipid level changes effected by HFHS diet and reverted them to levels observed with the control diet. To identify the magnitude of the changes in lipids after treatment with GSM in OVX rats, the FC in the HGHS + GSM diet group with respect to HFHS diet and control + GSM with respect to the control diet were calculated (Table 1 and Table S2). The majority of lipids were more affected by HFHS diets than GSM feeding. Of the 29 lipids, only 2 increased in relative intensity, while the remaining 27 decreased under the HFHS diet. The up-regulated lipids include two TGs (56:10, 60:12) containing long polyunsaturated fatty acids, and down-regulated lipids include six diglycerides (DGs), two lysophosphatidylcholines (LPCs), fifteen PCs, and four TGs. In OVX rats fed with HFHS + GSM, eleven lipids that had been down-regulated by HFHS were increased in relative intensity by >1-fold when compared with the HFHS diet. Treatment with GSM in rats fed with the HFHS diet resulted in significant increases in FC value for LPC (20:5) and TG (54:7), while PC (40:4) significantly decreased. Similarly, GSM treatment in the control group induced an increase in relative intensities of fourteen lipids by >1-fold when compared with the control. A significant increase in FC was noticed in two DGs (47:10, 49:10), two LPCs (16:1, 20:5), four PCs (32:2, 34:3, 36:6, 38:8), and all TGs, with exception of TG (48:1), when compared with the control diet. Conversely, GSM treatment resulted in significant decreases in relative intensity of four DGs (38:5, 38:6, 47:9, 49:12) and four PCs (34:4, 37:4, 38:7, 40:4). 

**Figure 3:**
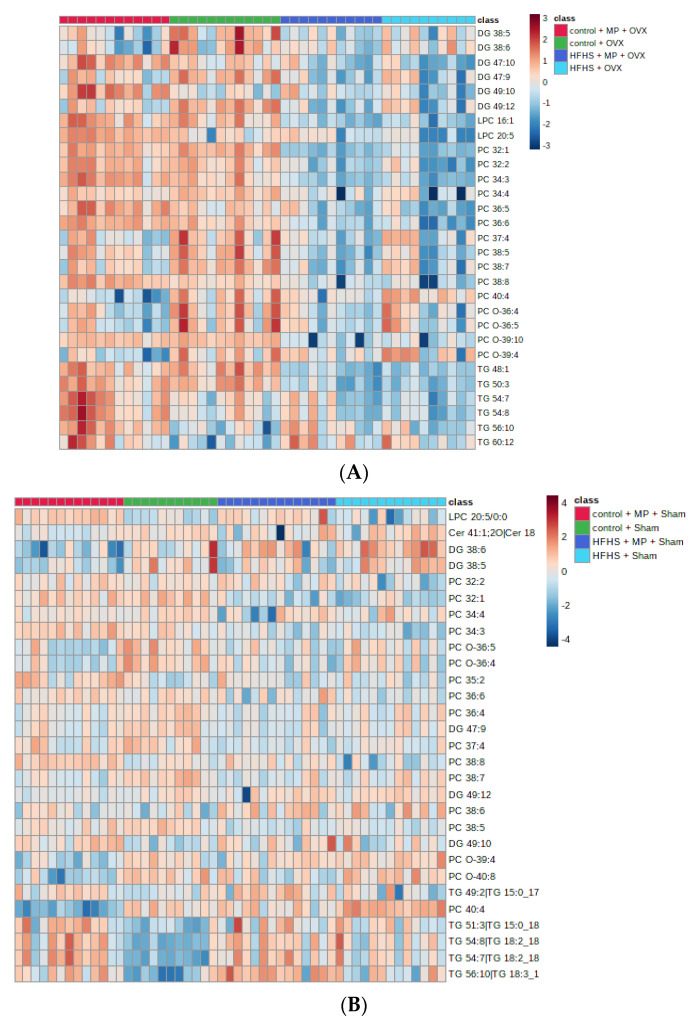
The heatmap showed the relationship between the comparison of contents of lipids in OVX (**A**) and sham (**B**); MP = greenshell mussel powder included in the diet. Each colour block corresponds to the value of relative content of each lipid.

The OPLS-DA was also performed on the dataset to identify lipids affected by HFHS and GSM in sham rats. Ultimately, 29 lipids were identified based on VIP > 1.0 and FDR < 0.05, as displayed in Table 2 and Table S3. Of these 29 lipid molecules, 22 lipids were commonly changed among OVX and sham rats. To identify the individual changes in the identified lipids, the FCs of these lipids were calculated for each diet group with respect to their control group. Similar to OVX rats, the relative intensity of all lipids, with exception of three TGs (54:7, 54:8, 56:10), was decreased under the HFHS diet when compared with the control diet group. In respect to down-regulated lipids, GSM treatment induced increases in the relative intensity of nine species with FC above 1, among which LPC 20:5 showed a significant increase. In contrast, two DGs (38:5, 38:6) and two PCs (40:4, O-40:8) showed significant decreases by GSM feeding when compared with the HFHS diet group. A similar trend was observed in the control diet; however, GSM treatment yielded more lipids with significant differences. The lipid biomarkers of the four diet groups are shown as a clustering heatmap in Figure 3A,B.

**Figure 4:**
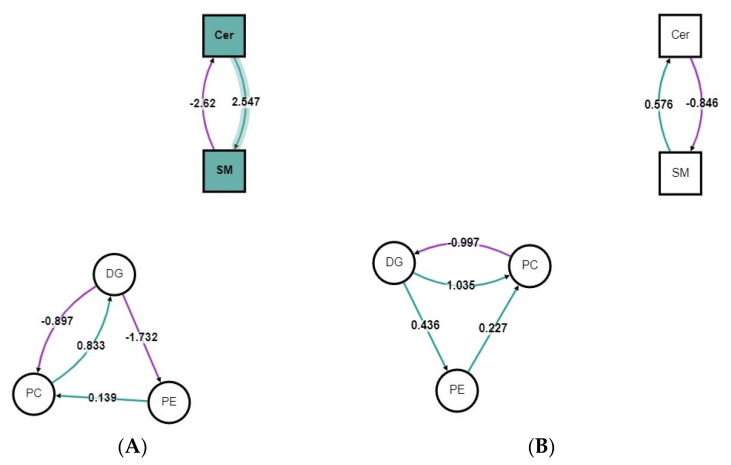
BioPAN lipid networks. Lipid network graphs exported from BioPAN for the HFHS diet vs. control (**A**) and the HFHS + GSM diet vs. HFHS diet (**B**) in OVX rats. Green nodes correspond to active lipids and green shaded arrows to active pathways. Reactions with a positive Z score have green arrows, while negative Z scores are coloured purple.

The lipid network at the lipid subclass level for the OVX rats is presented in Figure 4A for the HFHS diet group and Figure 4B for the HFHS + GSM diet group. Biological alterations usually occur in a complex set of changes over hundreds of lipid molecular species, rather than at the level of single lipid molecular species. Therefore, the lipidomic datasets including all the detected lipids species were uploaded in the BioPAN. The result showed that reactions using diacylglycerol (DG) as a substrate were suppressed in both HFHS versus control and HFHS + GSM versus HFHS diets, as shown in Figure 4. Diet-specific differences in lipid metabolism were shown by BioPAN, with the reaction pathway results of the HFHS diet indicating active metabolism, leading to the accumulation of sphingomyelin (Z-score = 2.547), opposite to the catabolic metabolism of SM observed in the HFHS + GSM diet (Z-score = 0.576). Similar findings were observed on the lipidomic profiles of sham rats. 

### Metabolomic Profile Change in Rats Fed with HFHS Diet and GSM

**Figure 5:**
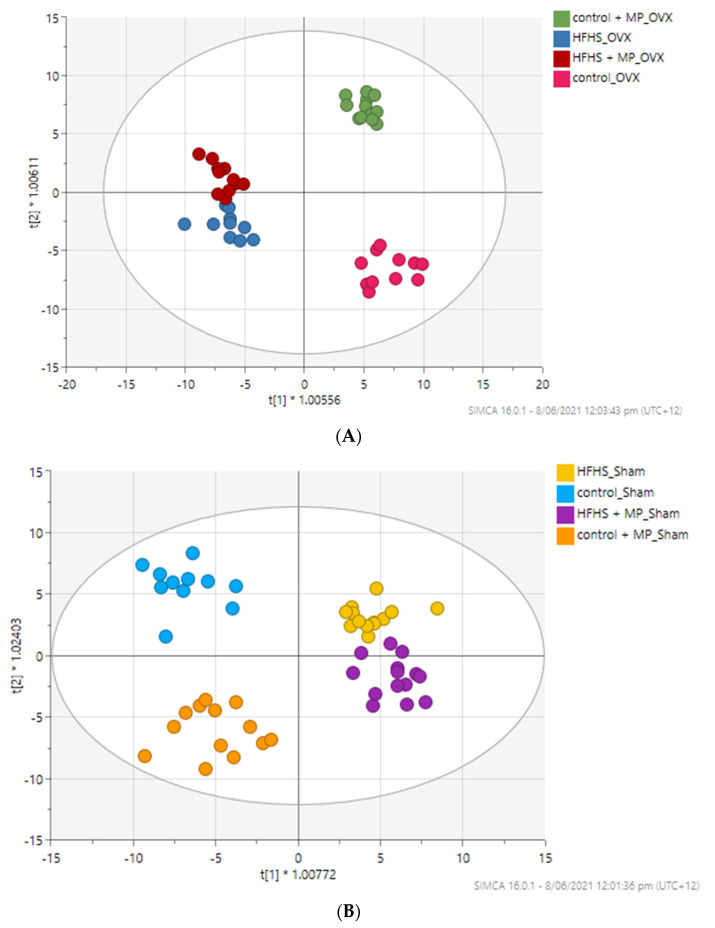
OPLS-DA analysis for metabolomic differences between diet groups showing good separation and robust modelling for sham and OVX rats; MP = greenshell mussel powder included in diet. Each circle represents the metabolite profile of single rat. (**A**) OPLS-DA scoring plot showing discrimination between diets groups in OVX rats (*n* = 44); R2X (cum) = 0.445, R2Y (cum) = 0.891, Q2 (cum) = 0.572. Cross-validated ANOVA *p*-value = 5.30302 × 10^−5^. (**B**) OPLS-DA scoring plot showing discrimination between diets groups in sham rats (*n* = 51); R2X (cum) = 0.307, R2Y (cum) = 0.624, Q2 (cum) = 0.401. Cross-validated ANOVA *p*-value = 1.32627 × 10^−4^.

A total of 525 features in negative ionisation and 604 in positive ionisation were detected by metabolomics. After filtration and removal of noise and unstable compounds, 422 features (including 383 unknown and 39 known metabolites) in positive and negative ions were combined into a single table for statistical analysis. PCA was applied to analyse the distribution of the polar metabolites among the four diet groups in OVX and sham rats (Figure S2). Overall, PCA showed separation between the HFHS and control diet in OVX rats, although it was less clear in sham rats. The OPLS-DA score plots between the diet groups are shown in Figure 5A,B. The model was validated with fitness R2X cum and R2Y cum values of 0.445 and 0.891, respectively, and with a predictability Q2 cum value of 0.572 in OVX rats. The R2X and R2Y cum in sham rats were 0.307 and 0.624, respectively, with predictability Q2 cum values of 0.401.

Using the OPLS-DA model, major metabolites significantly contributing to discrimination between the diet groups were selected based on both the VIP value > 1.0 and FDR < 0.05. In OVX rats, we identified 125 features fitting these criteria, of which eight were identifiable: cholic acid, serine, threonine, lysine, valine, pipecolic acid, histidine, and 3- methylcytidine. The identified metabolites are described in Table 3 and Table S4. Five showed increased levels under the HFHS diet. However, the levels of three of them (lysine, valine, and pipecolic acid) were regulated by GSM treatment with FC < 1. Further, all the metabolites were decreased by GSM treatment in the control diet, except for 3-methylcytidine.

In sham rats, we observed a total of 80 features based on VIP > 1 and FDR < 0.05, of which four were known metabolites: 3-hydrocybutric acid, valine, cholic acid, and tryptophan (Table 4 and Table S5). Two metabolites, 3-hydrocybutric acid and tryptophan, significantly decreased under the HFHS diet as compared with the control diet, while the level of cholic acid significantly increased. However, GSM treatment decreased the relative intensity of up-regulated metabolites valine and cholic acid when compared with HFHS, while down-regulated tryptophan indicated a slightly higher intensity with FC > 1 after GSM treatment, although these changes were not statistically significant. In respect to the control diet, GSM treatment induced an increase in the relative intensity of 3-hydrocybutric and cholic acid with an FC value of 1. Similar to OVX rats, a significant decrease in FC value < 1 was noted for valine.

A summary of pathways highlighted as being affected through feeding with HFHS diet and GSM in our metabolomic analysis is given in Table S6. Synthesis and degradation of ketone bodies, butanoate, and tryptophan metabolism was perturbed by HFHS diet in the sham group, along with changes occurring in primary bile acid biosynthesis in both the OVX and sham group. Further, significant changes in histidine and beta-alanine metabolism were observed by GSM treatment in OVX rats fed with the HFHS diet. Biosynthesis of valine, leucine, and isoleucine was significantly altered by GSM treatment in the control group in both OVX and sham rats.

The relationships between inflammatory and OA markers with identified lipid and metabolites were assessed. In this study, no significant association was observed for lipids, but tryptophan, which decreased under the HFHS diet, showed a positive association with CTX-1 and CTX-2 and was adversely associated with the inflammatory markers tumour necrosis factor-alpha (TNF-α) and monocyte chemotactic protein (MCP) (data not shown).

## 3. Discussion 

In this study, we revealed the effects of the HFHS diet and GSM supplementation on plasma lipid and polar metabolites of rats subjected to OVX or sham surgery using MS-based metabolomic techniques. Twenty-nine plasma lipids were identified and closely associated with the HFHS diet and/or GSM powder treatment. Most of the identified lipids were DG, PC, or TG, which were decreased by the HFHS diet, while TG containing long chain and highly unsaturated fatty acids such as TGs (56:10, 60:12) were increased. Some of the lipids significantly altered by the HFHS diet were recovered to normal diet levels by inclusion of GSM in the HFHS diet, although it was not an absolute recovery pattern. 

The metabolomic findings mainly centered on amino acid metabolism. Eight and four metabolites were identified in the OVX and sham rats, respectively. Feeding an HFHS diet to OVX rats resulted in an increase of a subset of two essential amino acids, lysine and valine, and one metabolite pipecolic acid; these were downregulated by adding GSM to the HFHS diet. Threonine was slightly decreased by the HFHS diet and then increased with GSM inclusion. In sham rats, 3-hydrocybutric acid and tryptophan were significantly reduced by the HFHS diet, while cholic acid increased. Among these altered metabolites, cholic acid and tryptophan were restored to normal levels by GSM.

Although this is the first study that investigated the changes in the plasma lipidome and metabolite profiles of HFHS diet-induced obesity in OVX rats, previous metabolomic analyses have shown altered metabolism in rodents fed with a high-fat diet as a model of MetOA. Mice fed a high-fat diet for 18 weeks exhibited a distinct lipidomic profile, in which lysoPCs (20:4, 17:0) and PC (36:2) were longitudinally and exclusively upregulated, and these biomarkers were able to predict the risk of OA induced by the high-fat diet [5]. Gowda et al. [15] recently reported the alteration of plasma lipids in rats with high fat diet-induced obesity. After 8 weeks of high-fat diet feeding, PC and TG containing PUFA such as PCs (32:1, 43:4, 32:4, 36:5) and TGs (52:6, 54:7) were significantly decreased in rats. In line with these studies, we observed a decline in PCs (32:1, 34:4, 36:5) and TG (54:7) in both OVX and sham rats fed with the HFHS diet. GSM treatment increased the level of some PC and LPC species that had been downregulated by the HFHS diet. As phospholipids are a major lipid class in the GSM lipid fraction [16], this indicates a potential biochemical role for GSM phospholipids that may regulate the concentration or synthesis of PC and LPC under HFHS diet conditions. However, the effect was not robust for most of the lipids; this is likely because of the fact that the lipid proportion of the whole GSM powder was too low in quantity in the diet to demonstrate the effect. Our result demonstrated alterations in the levels of three PCs (36:5, 36:6, 38:8), and lysoPC (20:5) under the HFHS diet which then were ameliorated after GSM treatment. Although our study did not assess the correlation between these lipids and cartilage damage to support their involvement in OA pathogenesis, there are a few studies investigating changes in lipid metabolites and their contribution to OA pathogenesis. As mentioned earlier, PC (36:2) and lysoPCs (20:4,17:0) were altered by a high-fat diet and showed strong associations with cartilage loss and OA severity in mice [5]. In a rat model of surgically-induced OA, levels of PCs (36:2, 38:7) and SM (d34:1) were positively correlated with cartilage damage [17]. It should be noted the reported results of studies investigating PCs and lysoPCs in OA have not been inconsistent, possibly owing to the differences in metabolic changes of PCs and lysoPCs in different subtypes of OA.

The TG levels showed different patterns of change depending on the length and degree of saturation of acyl chains. An increase in TGs (56:10, 60:12), but decrease in TGs (48:1, 50:3, 54:7, 54:8), was observed in OVX rats fed with the HFHS diet. Liu et al. demonstrated an increase in TG containing PUFA in rats with obesity induced by the high-fat diet for 3 weeks [18]. A prolonged high-fat diet induces adaptive adjustments in lipid metabolism in the liver such as compensatory synthesis of endogenous PUFA, while suppressing the formation of lipoproteins that transport the de novo synthesized PUFA to peripheral organs. This leads to the accumulation of PUFA in liver and plasma [19]. The decline in DG presented in the current study has not been observed elsewhere, with one study reporting increased circulating DG levels in female mice fed with a high-fat diet [20]. These contradictory findings may be due to the differences in animal species, feeding duration, age, and particularly diet composition. In addition, endogenous lipid biosynthesis related to bio-conversion mechanisms may impact the resultant circulating lipid profile.

Sphingolipids play an important role in cell growth, differentiation, apoptosis, and vital signal transduction pathways. A high-fat diet promotes de novo sphingolipid synthesis, resulting in the elevation of sphingomyelin (SM) and ceramide (Cer) [21] in plasma, adipose tissue, and liver [22]. We detected 22 SM and 3 Cer in the lipidomic analysis, although these lipids, with the exception of Cer 41:1, were not among the major lipids contributing to discrimination between the diet groups. Most of the detected SMs showed increases with the HFHS diet, which is in agreement with the lipid pathway analysis showing a shift towards the formation of SM. High-fat diet-induced elevation in SM level has been linked to sphingomyelin synthase 2 (SMS2) activity and overexpression [23]. However, the increased level of SM was higher than Cer in our study. It has been suggested that, in a state of elevated levels of SM compared with Cer, SM can serve as a pool for rapid ceramide generation [21]. Another possibility for not observing high amounts of Cer in this study could be related to the use of plasma rather than tissue samples; accumulation of Cer has been reported in non-adipose tissues of obese humans and rodents, which are responsible for the development of insulin insensitivity and related-metabolic dysregulation [24]. Further studies are required to investigate the mechanisms associated with decreased levels or inhibition of Cer synthesis under HFHS diets.

In vitro evidence using chondrocytes or explants from rabbit articular cartilage demonstrated that a high dose of C2-Cer induced apoptosis and up-regulated matrix metalloproteinase activity [25]. Higher concentrations of Cer species were observed in the synovial fluid of late-stage OA patients, implying the involvement of Cer species in OA progression [26]. The exact function of SM and Cer species in the pathophysiology of OA induced by obesity is still unknown; however, the reaction pathway results in the current study showed GSM treatment suppressed SM synthesis and significantly reduced cartilage degradation in the rat trial [8]. This may suggest a potential role for GSM as a therapeutic or preventative intervention for high-fat-induced pathologic conditions by controlling and normalizing abnormal SM metabolisms. However, further mechanistic studies need to be conducted to dissect the mechanisms underlying high fat diet-mediated sphingolipid metabolism in OA and the impact of GSM treatment.

Additional metabolites were identified in our study as potential biomarkers that may explain the impact of HFHS diet and GSM treatment. In OVX rats, most of the metabolites including cholic acid, serine, lysine, valine, and pipecolic acid were increased by the HFHS diet, while threonine, histidine, and 3-methylcytidine were down-regulated. In sham rats, 3-hydrocybutric acid and tryptophan decreased under the HFHS diet, while valine and cholic acid showed the same trend as OVX rats under the HFHS diet. Consistent with previous reports [27,28], our results showed that the levels of the branched-chain amino acids (BCAAs) lysine, and particularly valine, were elevated by the HFHS diet. Meanwhile, 3-hydrocybutric acid, a catabolic intermediate of valine, declined with the HFHS diet. Higher levels of BCAA are directly involved in insulin resistance via inhibiting the activity of AMP-activated protein kinase and, subsequently, contributing to metabolic dysregulation [29]. Additionally, liver dysfunction caused by high-fat diet has been found to result in the accumulation of pipecolic acid, which is a minor metabolite of lysine, in the serum of obese mice [30]; this is consistent with our findings. Our data also showed that serine and threonine were increased by the HFHS diet in OVX rats, in whom myocardial hypertrophy and renal tubular epithelium degeneration occurred at ≥2X the incidence rate of OVX rats on a normal diet [31]. These data verify findings of a previous study [32], which demonstrated abnormal changes in serine and threonine in conditions such as myocardial injury. Further, alterations in amino acids such as tryptophan, alanine, valine, and histidine, along with increases in relative concentrations of n-butyrate and α-hydroxy-n-butyrate, have been demonstrated in the urine of type 2 diabetes mellites animal models and human patients. Moreover, increased urinary β-hydroxybutyrate was also identified to increase with age in diabetic rats [33]. The rats used in the current study were aged and oestrogen-deficient in addition to eating a chronic HFHS diet, resulting in significant metabolic disorder. Thus, these metabolites may eventuate as biomarkers for the detection or prediction of other disease states.

Elevated bile acid is caused by hepatic dysfunction. Cholic acid as a major primary bile acid is synthesised from cholesterol in the liver and has implications in lipolysis, cholesterol catabolism, and overall regulation of lipid metabolism [34]. A high fat diet leads to the accumulation of triglycerides and cholesterol in rat livers, which upregulates the bile acid synthetic enzymes while downregulating their export and transportation. These changes result in bile acid deposition in the liver, leading to steatohepatitis [35]. Cholic acid was elevated by the HFHS diet in our study, and these rats in both OVX and sham groups had approximately double the incidence of periportal bile ductular hyperplasia in the liver compared with their normal diet counterparts [31]; this is supported by previous findings [36]. Therefore, the increased cholic acids may be partly explained by prolonged consumption of a high fat diet, causing dysregulation in bile acid hepatic homeostasis. 

The levels of some of up-regulated amino acids including lysine, valine, and pipecolic acid were decreased and partially recovered with GSM treatment. These results suggest that GSM treatment might partially improve the impaired amino acid metabolism induced by HFHS feeding. The GSM components that contributed to the potential efficacy observed in the OA disease model are as yet unknown; nevertheless, numerous bioactive compounds have been identified within GSM that possess anti-inflammatory, antioxidant, anti-hypertensive, antibacterial, and antithrombin effects, mainly derived from its peptides, carbohydrates, and lipids [37]. Analysis of bioactive peptides revealed a high level of amino acids, such as glycine, valine, lysine, isoleucine, and alanine, which might have provided the observed effects.

With regard to the estrogen-deficient condition, it was noted that the majority of lipids commonly changed among OVX and sham rats shared similar trends. This may suggest that, in this model, the plasma lipid profiles in rats were predominantly influenced by feeding conditions rather than estrogen deficiency. In a previous animal model, ovariectomy caused abnormality in lipid metabolism along with significant increases in body weight gain and fat accumulation in the liver and blood. However, it was found that the quantity and types of dietary fatty acids can affect these, as a diet with a high proportion of MUFA and PUFA can interfere with the fatty acid metabolism pathways and adjust the adverse effect of OVX [38]. With respect to GSM treatment in the current study, the control diet group presented accentuated lipidomic responses to GSM consumption, while the HFHS diet group showed a more tapered shift, possibly owing to homeostatic mechanisms in response to prolonged feeding of HFHS. 

Although the results presented here are not sufficient to establish a direct contribution of the altered metabolites to OA pathogenesis, most of these lipids and metabolites have been shown to be significantly altered in plasma in other obesity-related disorders [15,18]. Moreover, the data were collected at only one time point and, in this study, no strong correlation was observed between these lipids and polar metabolites and biomarkers of cartilage degradation or bone resorption, indicating that these metabolites might be more closely connected to HFHS-induced obesity rather than to OA. The metabolomic profile of synovial fluid or cartilage tissue during disease-related alterations, as well as the metabolomic profile of plasma during the early stages of OA when the biomarkers of cartilage degradation are more prominent, will provide more precise information regarding the molecular changes of the joint microenvironment in MetOA and possible mechanisms of GSM to treat or prevent joint diseases. Regardless, this is the first study to report the combined effect of HFHS diet, OVX, and GSM treatment on plasma lipid and metabolites of rats, and provides important novel information about the individual and combined effects of these factors. 

## 4. Methods

### 4.1. Experimental Methods

**Figure 6:**
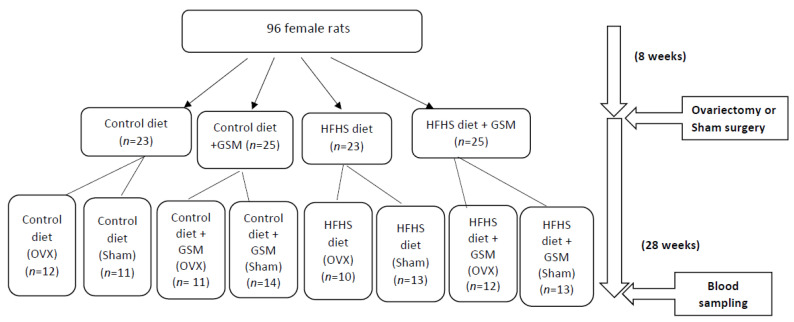
Overview of the study design. Rats were randomized by maternal parentage, bone mineral density, and body weight into groups of control diet (5% sugar, 5% fat, 15% protein from casein), control + GSM diet (5% sugar, 5% fat, 15% protein from 2:1 casein: GSM), HFHS diet (30% sugar, 30% fat, 15% protein from casein), or HFHS + GSM diet (30% sugar, 30% fat, 15% protein from 2:1 casein: GSM) diet. Eight weeks into the study, approximately half of the rats in each group underwent sham surgery (ovaries left intact), while the other half underwent ovariectomy (OVX), after again being randomized by bone mineral density and body weight. Blood samples were collected after 36 weeks.

The methodology used for the animal study was as described elsewhere [8], and the overall experimental design is presented in Figure 6. In brief, 96 female Sprague–Dawley rats were divided into four experimental diets: control, control supplemented with GSM (control + GSM), high fat/high sugar (HFHS), or high fat/high sugar enriched with GSM (HFHS + GSM). The proximal composition of diets can be found in Figure 1 and Table S1. After eight weeks on an experimental diet, half of the rats underwent bilateral ovariectomy (OVX) and the rest of the animals underwent a sham surgical procedure. The test diet regimen continued through the end of the experiment. After 36 weeks, blood samples were collected from all rats by cardiac puncture into EDTA-anticoagulated tubes. The EDTA tubes were centrifuged at 1050× *g*, the plasma was removed, and samples were kept at −80 °C until analysis. This study was performed in full compliance with the Massey University Animal Ethics committee (approval number 16/112). 

### 4.2. Metabolomic Analysis

#### 4.2.1. Chemicals and Reagents

Solvents and chemicals used for sample preparation, mobile phase, and LC–MS analysis (chloroform, methanol, acetonitrile isopropanol, and formic acid) were purchased from Thermo Fisher Scientific (Auckland, New Zealand). Milli-Q^®^ ultrapure water was purchased from Merck Millipore (Bedford, MA, USA). Ammonium formate (Fluka™, HPLC grade) was obtained from Sigma-Aldrich (Auckland, New Zealand). Lipid internal standard 1-palmitoyl(D31)-2-oleoyl-sn-glycero-3-phosphoethanolamine (16:0 d31-18:1-PE) was obtained from Avanti^®^ (Avanti Polar Lipids, Alabaster, AL, USA). All chemicals were of LC–MS grade except chloroform, which was of analytical grade.

#### 4.2.2. Sample Preparation 

Plasma samples were thawed at 4 °C overnight and then vortexed. For lipid extraction, 100 μL of plasma was transferred into a 2 mL Eppendorf tube and mixed with 800 μL of − 20 °C extraction buffer (CHCl_3_:MeOH 1:1, containing approximately 1.6 mg/L internal standards D_5_-L-tryptophan, D_10_-leucine, D_2_-tyrosine, and D_7_-alanine), vortexed for 30 s, and stored at −20 °C for 60 min. Subsequently, 400 μL of water was added, and the mixture was vortexed for 30 s and centrifuged for 10 min at 13,663× *g* at 4 °C (Eppendorf Centrifuge 5427 R, Germany). Blank samples were prepared using the same procedure, except that the plasma was replaced with 100 μL of MilliQ water. The pooled lipid QC samples were prepared by combining 30 μL of the lower phase from each sample in a new tube. The pooled QC samples were vortexed, aliquoted into multiple Eppendorf tubes, and evaporated to dryness under a stream of nitrogen at room temperature (Techne^®^ Sample Concentrator) in the same manner as the rest of the samples. The dried samples were stored at −80 °C until further analysis.

Polar metabolites were extracted from the plasma samples following a monophasic extraction [39]. Briefly, 50 μL of plasma was placed into an Eppendorf tube, to which was added 450 µL of pre-chilled acetonitrile/water (9:1 *v*/*v*). The mixture was vortexed (60 s at 30 Hz) and then centrifuged for 10 min at 13,663× *g* at 4 °C (Eppendorf Centrifuge 5427 R, Germany), and 200 µL of extract was placed into an HPLC vial for analysis. All samples were stored at − 80 °C for subsequent LCMS analysis of polar metabolites using HILIC chromatography. For QC samples, 100 µL of each extract was taken to form a pooled QC and added to HPLC vials for analysis.

#### 4.2.3. Instruments and Conditions

Lipid extracts were analysed using a Shimadzu LCMS-9030 mass spectrometer equipped with a Shimadzu Nexera-x2 Ultra Performance Liquid Chromatography^®^ (UPLC) system by injecting 2 µL onto a Waters CSH-C18 column (2.1 × 100 mm, 1.7 µm particle size). Samples were held in the autosampler at 20 °C and the column oven was held at 60 °C. Lipids were eluted over a 15 min gradient with a flow rate of 400 μL/min. The mobile phase was a mixture of water/acetonitrile/isopropanol (5:3:2 *v*/*v* containing 10 mM ammonium formate) (solvent A) and water/acetonitrile/isopropanol (1:9:90 *v*/*v* containing 10 mM ammonium formate) (solvent B). The gradient elution programme was as follows: 10–45% B (0–2.7 min), 45–53% B (2.7–2.8 min), 53–65% B (2.8–9 min), 65–89% B (9–9.1 min), 89–92% B (9.1–11 min), and finally to 100% B (11–11.1 min) and held for 0.8 min (11.1–11.9 min) before returning to 10% B (11.9–12 min) and held to re-equilibrate until 15 min [40]. The mass spectrometer was operated in positive ionisation mode, measuring full MS1 spectra from 250 to 1250 *m/z* across the entire chromatogram, and collecting data independent acquisition (DIA) data in 20 *m/z* windows from 300 to 1100 *m/z*, with a 0.6 s cycle time and collision energy of 25 normalised collision energy units. The source voltage was + 4.0 kV, with a nebulising gas flow of 2.0 L/min, heater gas flow of 10 L/min, interface temperature of 300 °C, drying gas flow of 10 L/min, desolvation line temperature of 250 °C, and heater block temperature of 400 °C. All drying and collision gasses used were nitrogen.

Polar metabolite extracts were analysed using a Shimadzu LCMS-9030 mass spectrometer equipped with a Shimadzu Nexera-x2 UHPLC system. Polar metabolites were measured by injecting 5 µL onto a Thermo Accucore HILIC column (2.1 × 100 mm, 2.6 µm particle size). Samples were held in the autosampler at 4 °C and the column oven was held at 30 °C. Metabolites were eluted over a 23 min linear gradient with a flow rate of 400 μL/min. The mobile phases were water containing 10 mM ammonium formate (solvent A) and acetonitrile containing 0.1% formic acid (solvent B). The mass spectrometer was operated in both positive and negative ionisation modes, measuring full MS1 spectra from 70 to 1000 *m/z* across the entire chromatogram, and collecting DIA data in 20 *m/z* windows from 70 to 900 *m/z*, with a 0.6 s cycle time and collision energy of 25 normalised collision energy units. The source voltage was +4.0 kV, with a nebulising gas flow of 2.0 L/min, heater gas flow of 10 L/min, interface temperature of 300 °C, drying gas flow of 10 L/min, desolvation line temperature of 250 °C, and heater block temperature of 400 °C. All drying and collision gasses used were nitrogen.

#### 4.2.4. Data Processing and Statistical Analysis 

Data were captured and converted to centroid MZML format using the Shimadzu file converter and imported into MS-Dial software for peak detection, gap filling, alignment, and noise elimination. Identification of the aligned peaks was performed using the DIA MS/MS spectral. For lipidomic, features were searched against the built-in lipid library containing 257,000 in silico generated MS/MS lipid fragmentation spectra, while for metabolites, these were searched against the MS/MS public library containing 13,303 unique compounds [41]. The resultant peak intensity table underwent run-order correction and normalization using pooled QC samples and the locally weighted scatterplot smoother (LOWESS) regression model. Ultimately, features with an average of QC-to-blank sample ratio of < 5 and CV of 30% within the pooled QC samples were removed. In total, the full datasets for 96 rat samples were included in the lipidomic and 95 for metabolomic analysis as data from polar metabolites were not available for one sample.

Statistical analysis of the metabolite and lipid data was conducted using SIMCA version 16.0.1. software (Umetrics, Umea, Sweden). Multivariate analyses including PCA (principal component analysis) and OPLS-DA (orthogonal partial least squares discriminant-analysis) were conducted to obtain information on differences in lipid and polar metabolite profiles between experimental diet groups. OPLS-DA has been used by previous studies to discriminate two or more groups (classes) using multivariate data [42]. In our study, the OPLS-DA models were validated using the predictive ability of the model (Q2) and the analysis of variance testing of cross-validated predictive residuals (CV-ANOVA) [43], which is a diagnostic tool for assessing the reliability of OPLS models. The cross-validation was used to evaluate the robustness of the model. The features with values of VIP > 1 and using Benjamini–Hochberg false discovery rate (FDR) < 0.05 were considered to select the relevant lipid and polar metabolites. Subsequently, the relative intensity of the features was used to calculate the fold changes (FCs) in lipids and polar metabolites between the treatment groups and their control using Metaboanalyst version 4.0 (Figure S7) [44]. The metabolomic data were input into Metaboanalyst for pathway enrichment analysis utilizing the Kyoto Encyclopedia of Genes and Genomes (KEGG) platform to analyze the topological characteristics of highly correlated metabolic pathways [45].

Further, to visualise the quantitative lipidomic data from the perspective of biosynthetic pathways, lipidomic data were input into the BioPAN platform available at https://lipidmaps.org/biopan/ Accessed on 1 June 2021) to search the highly correlated metabolic pathways. BioPAN is a web-based tool on available at the LIPID MAPS^®^ Lipidomics Gateway, which enables combining lipid metabolism with a statistical analysis functionality. Nguyen et al. has originally described the statistical model used in BioPAN [46]. Briefly, BioPAN uses quantitative lipidomic data from two experiments (e.g., a condition of interest treated and a control condition) and calculates the statistical scores to identify the activated or suppressed pathways in the treated samples compared with the control set [47]. The statistical significance of the difference in the ions between the groups was determined using Student’s t-test and accepting significance at *p*-value < 0.05.

## 5. Conclusions

In summary, untargeted lipidomic and metabolomic analysis identified several lipids in glycerophospholipids, glycerolipids, and sphingolipids and amino acids altered by HFHS diet and GSM supplementation. Adding GSM recovered some of the metabolomic alterations under the HFHS diet with particularly robust responses in rats fed a control diet. Subsequent evaluation of lipid pathways revealed that sphingolipid metabolism may be affected by the HFHS diet, suggesting that increases in SM and Cer levels are potential causes of metabolic dysregulation under HFHS feeding, an effect that may be repaired by adding GSM to the diet. These results help provide a basis for the metabolic changes that occur in female-specific obesity and MetOA induced by an HFHS diet and ovariectomy, which highlight the need for additional tracking clinical studies on postmenopausal women.

## Figures and Tables

**Table 1:** Plasma lipid biomarkers affected by the HFHS diet or GSM treatment in OVX rats.

Lipids	Average Rt (Min)	Adduct Type	Fold Change		
			HFHS/ Control	HFHS + GSM/HFHS	Control + GSM/ Control
DG 38:5	8.227	[M+Na]+	0.54 *	0.76	0.49 *
DG 38:6	7.444	[M+Na]+	0.76	0.83	0.61 *
DG 47:10	6.276	[M+Na]+	0.74 *	1.09	1.2 *
DG 47:9	6.188	[M+Na]+	0.56 *	0.78	0.66 *
DG 49:10	7.184	[M+Na]+	0.89	1.12	1.34 *
DG 49:12	6.177	[M+Na]+	0.69 *	0.88	0.76 *
LPC 16:1	2.228	[M+H]+	0.33 *	0.96	1.19 *
LPC 20:5	1.994	[M+H]+	0.28 *	2.6 *	4.38 *
PC 32:1	6.065	[M+H]+	0.19 *	0.79	0.87
PC 32:2	5.506	[M+H]+	0.46 *	1.00	1.14
PC 34:3	5.647	[M+H]+	0.42 *	1.01	1.34 *
PC 34:4	5.442	[M+H]+	0.37 *	0.79	0.69 *
PC 36:5	6.268	[M+H]+	0.73 *	1.1	1.19
PC 36:6	5.639	[M+H]+	0.31 *	1.24	1.32 *
PC 37:4	6.613	[M+H]+	0.65	0.74	0.6 *
PC 38:5	6.264	[M+H]+	0.41 *	0.81	0.69
PC 38:7	6.181	[M+H]+	0.7 *	0.87	0.76 *
PC 38:8	5.685	[M+H]+	0.24 *	1.29	2.26 *
PC 40:4	7.786	[M+H]+	0.92	0.54 *	0.32 *
PC O-36:4	6.702	[M+H]+	0.66 *	0.8	0.59 *
PC O-36:5	6.567	[M+H]+	0.69	0.83	0.59 *
PC O-39:10	6.912	[M+H]+	0.24 *	0.76	0.87
PC O-39:4	7.076	[M+H]+	0.97	0.81	0.62 *
TG 48:1	10.944	[M+NH_4_]+	0.2 *	0.97	0.84
TG 50:3	10.844	[M+NH_4_]+	0.25 *	1.08	1.03
TG 54:7	10.732	[M+NH_4_]+	0.62 *	1.4 *	1.88 *
TG 54:8	10.638	[M+NH_4_]+	0.66 *	1.36	2.03 *
TG 56:10	10.589	[M+NH_4_]+	1.07	1.36	2.22 *
TG 60:12	10.677	[M+NH_4_]+	1.46 *	1.16	1.94 *

Lipids were determined using the VIP value > 1 and FDR < 0.05 from the OPLS-DA model. Fold change was calculated by dividing the mean of the peak intensity of each lipid from each of the two groups. * Lipids showing significant differences (*p*-value < 0.05) between groups as determined by Student’s *t*-test.

**Table 2:** Plasma lipid biomarkers affected by the HFHS diet or GSM treatment in sham rats.

			Fold Change		
Lipids	Average Rt (min)	Adduct Type	HFHS/ Control	HFHS + GSM/HFHS	Control + GSM/ Control
Cer 41:1	10.315	[M+H-H_2_O]+	0.54 *	0.63	0.69 *
DG 38:5	8.227	[M+Na]+	0.62 *	0.69 *	0.54 *
DG 38:6	7.444	[M+Na]+	0.91	0.74 *	0.61 *
DG 47:9	6.188	[M+Na]+	0.47 *	0.77	0.71 *
DG 49:10	7.184	[M+Na]+	0.91	1.02	1.2 *
DG 49:12	6.177	[M+Na]+	0.65 *	0.8	0.78 *
LPC 20:5	1.994	[M+H]+	0.41 *	1.58 *	3.14 *
PC 32:1	6.065	[M+H]+	0.21 *	0.97	0.89
PC 32:2	5.506	[M+H]+	0.4 *	1.18	1.05
PC 33:2	5.87	[M+H]+	0.47 *	0.88	1.27
PC 34:3	5.647	[M+H]+	0.44 *	1.01	1.19
PC 34:4	5.442	[M+H]+	0.27 *	0.74	0.68 *
PC 35:2	6.695	[M+H]+	0.68 *	1.03	1.34 *
PC 36:4	6.186	[M+H]+	0.57 *	0.79	0.78 *
PC 36:6	5.639	[M+H]+	0.28 *	1.06	1.08
PC 37:4	6.613	[M+H]+	0.49 *	0.83	0.8
PC 38:5	6.264	[M+H]+	0.38 *	0.86	0.77
PC 38:7	6.181	[M+H]+	0.65 *	0.84	0.78 *
PC 38:8	5.685	[M+H]+	0.26 *	1.43	2.16 *
PC 40:4	7.786	[M+H]+	0.71	0.63 *	0.32 *
PC O-36:4	6.702	[M+H]+	0.5 *	0.82	0.62 *
PC O-36:5	6.567	[M+H]+	0.49 *	0.84	0.63 *
PC O-39:4	7.076	[M+H]+	0.75 *	0.82	0.7 *
PC O-40:8	6.79	[M+H]+	0.71 *	0.77 *	0.63 *
TG 49:2	10.894	[M+NH_4_]+	0.52 *	1.4	1.35 *
TG 51:3	10.905	[M+NH_4_]+	0.96	1.05	1.47 *
TG 54:7	10.732	[M+NH_4_]+	1.1	1.11	2 *
TG 54:8	10.638	[M+NH_4_]+	1.12	1.04	1.8 *
TG 56:10	10.589	[M+NH_4_]+	1.09	1.3	1.87 *

Lipids were determined using the VIP value > 1 and FDR < 0.05 from the OPLS-DA model. Fold change was calculated by dividing the mean of the peak intensity of each lipid from each of the two groups. * Lipids showing significant differences (*p*-value < 0.05) between groups as determined by Student’s *t*-test.

**Table 3:** Plasma metabolite biomarkers affected by the HFHS diet or GSM treatment in OVX rats.

Metabolites	Average Rt(Min)	Adduct Type	Fold Change		
			HFHS/ Control	HFHS + GSM/ HFHS	Control + GSM/ Control
Cholic acid	2.572	[M+H]+	1.42 *	1.02	0.59
Serine	11.141	[M+H]+	1.28	1.06	0.65 *
Threonine	10.263	[M+H]+	0.94	1.21	0.58 *
Lysine	9.533	[M+H]+	1.37 *	0.95	0.90 *
Valine	6.481	[M+H]+	1.19	0.82 *	0.71 *
Pipecolic acid	9.534	[M+H]+	1.69 *	0.91	0.33 *
Histidine	8.167	[M-H]−	0.97	0.87 *	0.93
3-Methylcytidine	6.516	[M+H]+	0.83	0.99	1.45 *

Metabolites were determined using the VIP value > 1.0 and FDR < 0.05 from the OPLS-DA model. Fold change was calculated by dividing the mean of the peak intensity of each lipid from each of the two groups. * Metabolites showing significant differences (*p*-value < 0.05) between groups as determined by Student’s *t*-test.

**Table 4:** Plasma metabolite biomarkers affected by HFHS diet or GSM treatment in sham rats.

Metabolites	Average Rt (Min)	Adduct Type	Fold Change		
			HFHS/ Control	HFHS + GSM/ HFHS	Control + GSM/ Control
3- hydroxybutyric acid	6.863	[M-H]−	0.55 *	0.98	1.16
Valine	6.481	[M+H]+	1.03	0.80	0.63 *
Cholic acid	2.572	[M+H]+	1.38 *	0.42	1.23
Tryptophan	7.968	[M+H]+	0.80 *	1.06	0.91

Metabolites were determined using the VIP value > 1.0 and FDR < 0.05 from the OPLS-DA model. Fold change was calculated by dividing the mean of the peak intensity of each lipid from each of the two groups. * Metabolites showing significant differences (*p*-value < 0.05) between groups as determined by Student’s *t*-test.

## Data Availability

Data is contained within the article or supplementary material, the data presented in this study are available in https://www.mdpi.com/article/10.3390/metabo11110754/s1.

## Supplementary Materials

The following are available online at https://www.mdpi.com/article/10.3390/metabo11110754/s1, Figure S1: PCA scatter plot derived from lipidomic database, Figure S2: PCA scatter plot derived from metabolomic database, Figure S3. Flowchart of data analysis in metabolomics, Table S1. Composition of four experimental diet groups, Table S2. Lipids-OVX rats, Table S3. Lipids-Sham rats, Table S4. Metabolites-OVX rats, Table S5. Metabolites-Sham rats, Table S6: Description of the total number of compounds in the pathway among diet groups.
