# Supplementary material for: Mass Spectrometry-Based Metabolomic and Lipidomic Analysis of the Effect of High Fat/High Sugar Diet and GreenshellTM Mussel Feeding on Plasma of Ovariectomized Rats
Source: Metabolites. 2021 Oct 31;11(11):754. doi: 10.3390/metabo11110754 (PMC8622240; doi:10.3390/metabo11110754)
Supplement: Supplementary file 1 [file metabolites-11-00754-s001.zip › supplementary files.pdf]

Table S1. Composition of four experimental diet groups

Nutrient (%)	High fat/high sugar	High fat/high sugar+ GSM	Control	Control + GSM
Carbohydrate	30 (sucrose)	30 (sucrose)	5 (sucrose)	5 (sucrose)
Fat	30 (soy oil)	30 (49% from soy oil, 49% from lard, 1% from GSM)	5 (soy oil)	5 (84% from soy oil, 16% from GSM)
Protein	15 (casein)	15 (66% from casein, 33% from GSM)	15 (casein)	15 (66% from casein, 33% from GSM)

(A)

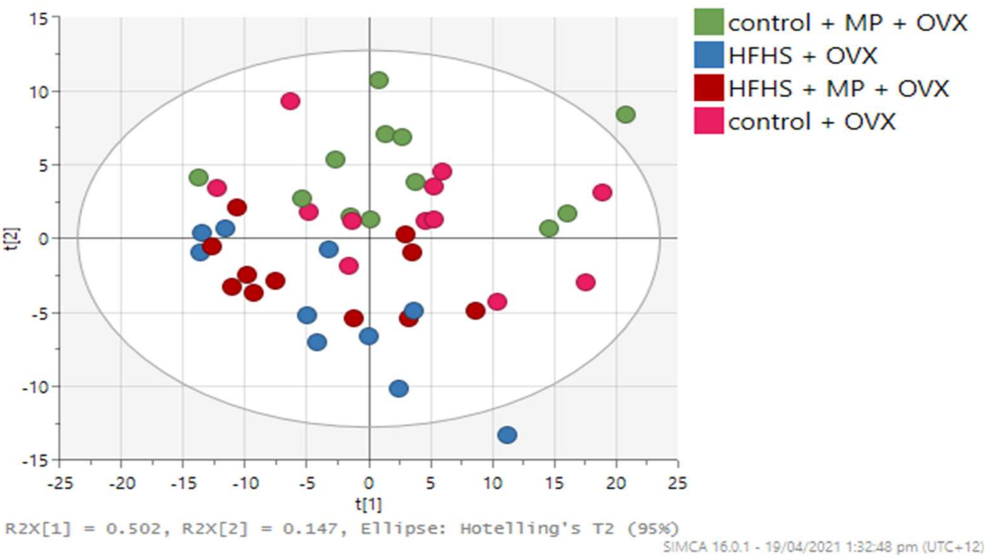

(B)

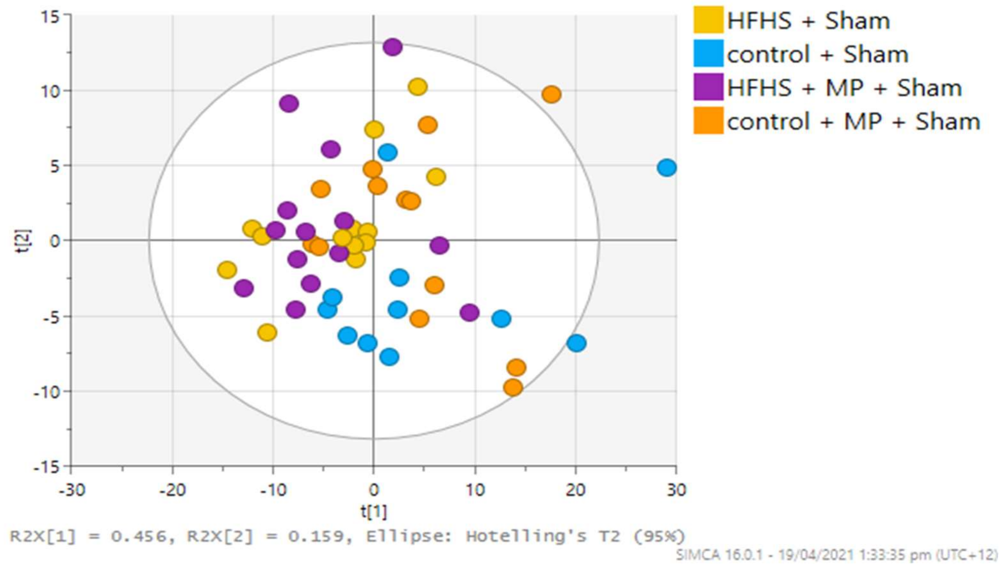

Figure S1: PCA scatter plot derived from lipidomic database. Each circle represents lipid profile of single rat. (A) PCA scoring plot showing variation of four diets groups in OVX rats (n=45), R2=0.869 and Q2=0.749. (B) PCA scoring plot showing variation of four diets groups in sham rats (n=51), R2=0.615 and Q2=0.544.

(A)

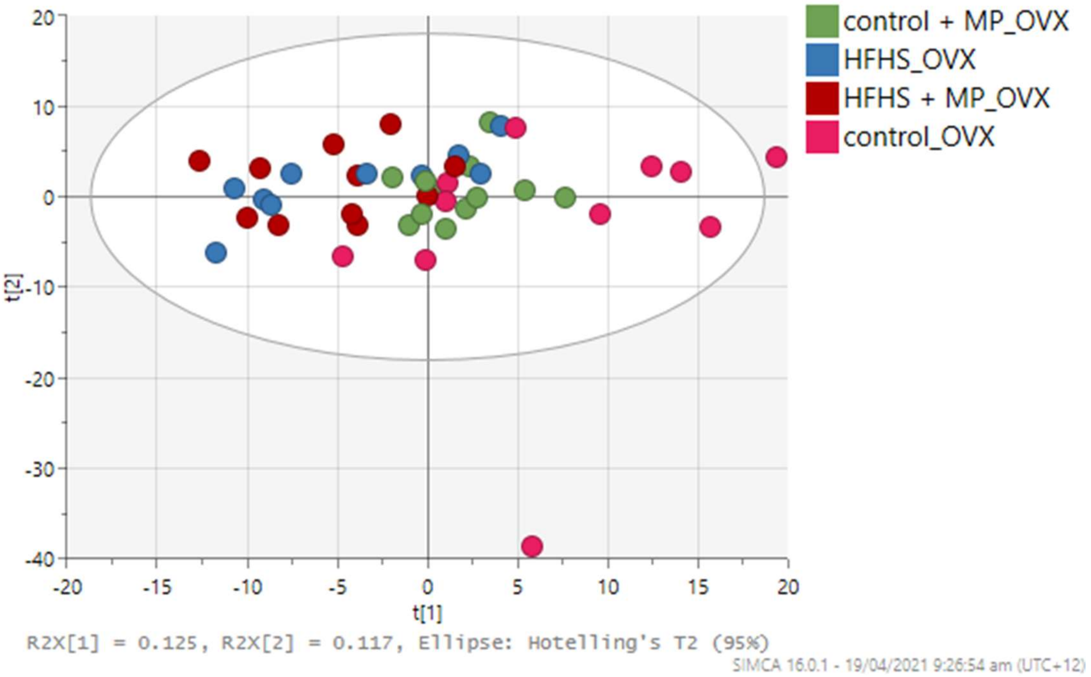

(B)

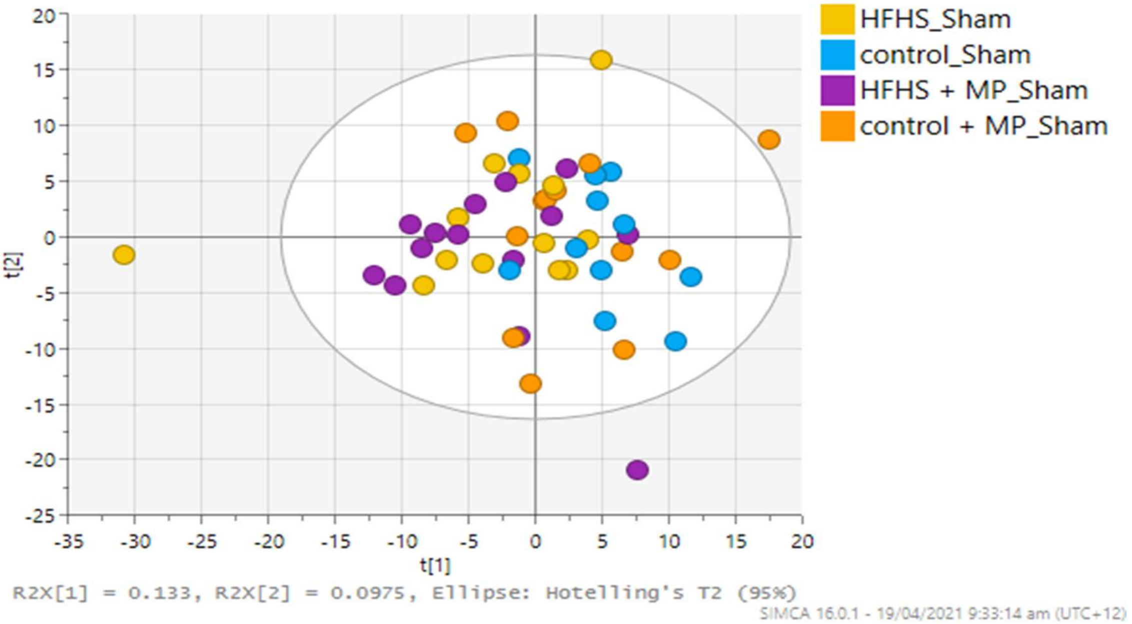

Figure S2: PCA scatter plot derived from metabolomic database. Each circle represents lipid profile of single rat. (A) PCA scoring plot showing variation of four diets groups in OVX rats (n=44), R2=0.65 and Q2=0.133. (B) PCA scoring plot showing variation of four diets groups in sham rats (n=51), R2=0.683 and Q2=0.232.

Table S6. Description of the total number of compounds in the pathway among diet groups.

HFHS vs control diet in OVX rats							
	Total Compound[1]	Hits[2]	Raw p[3]	-Log (p)	Holm adjust	FDR	Impact[4]
Primary bile acid biosynthesis	46	1	0.004733	2.3249	0.061526	0.061526	0
Biotin metabolism	10	1	0.024535	1.6102	0.29443	0.11663	0
Lysine degradation	25	2	0.026915	1.57	0.29606	0.11663	0
Aminoacyl-tRNA biosynthesis	48	5	0.038246	1.4174	0.38246	0.1243	0.16667
Valine, leucine and isoleucine degradation	40	1	0.058505	1.2328	0.52655	0.12676	0
Pantothenate and CoA biosynthesis	19	1	0.058505	1.2328	0.52655	0.12676	0
Glyoxylate and dicarboxylate metabolism	32	1	0.10461	0.98042	0.73227	0.1511	0.04233
Cysteine and methionine metabolism	33	1	0.10461	0.98042	0.73227	0.1511	0.02184
Sphingolipid metabolism	21	1	0.10461	0.98042	0.73227	0.1511	0
Valine, leucine and isoleucine biosynthesis	8	2	0.16819	0.77419	0.73227	0.21865	0
Glycine, serine and threonine metabolism	34	2	0.26919	0.56994	0.80757	0.31813	0.23069
Histidine metabolism	16	1	0.6455	0.1901	1	0.6455	0.22131
beta-Alanine metabolism	21	1	0.6455	0.1901	1	0.6455	0

HFHS+GSM vs HFHS diet in OVX rats							
	Total Compound[1]	Hits[2]	Raw p[3]	-Log (p)	Holm adjust	FDR	Impact[4]
Histidine metabolism	16	1	0.006228	2.2057	0.080959	0.040479	0.22131
beta-Alanine metabolism	21	1	0.006228	2.2057	0.080959	0.040479	0
Aminoacyl-tRNA biosynthesis	48	5	0.017514	1.7566	0.19265	0.063189	0.16667
Valine, leucine and isoleucine degradation	40	1	0.028826	1.5402	0.28826	0.063189	0
Pantothenate and CoA biosynthesis	19	1	0.028826	1.5402	0.28826	0.063189	0
Valine, leucine and isoleucine biosynthesis	8	2	0.029164	1.5352	0.28826	0.063189	0
Glycine, serine and threonine metabolism	34	2	0.25081	0.60066	1	0.46579	0.23069
Glyoxylate and dicarboxylate metabolism	32	1	0.41244	0.38464	1	0.53617	0.04233
Cysteine and methionine metabolism	33	1	0.41244	0.38464	1	0.53617	0.02184
Sphingolipid metabolism	21	1	0.41244	0.38464	1	0.53617	0
Primary bile acid biosynthesis	46	1	0.60804	0.21607	1	0.7186	0
Biotin metabolism	10	1	0.7441	0.12837	1	0.75483	0
Lysine degradation	25	2	0.75483	0.12215	1	0.75483	0

control+ GSM vs control diet in OVX rats							
	Total Compound[1]	Hits[2]	Raw p[3]	-Log (p)	Holm adjust	FDR	Impact[4]
Glycine, serine and threonine metabolism	34	2	0.001981	2.7031	0.025757	0.01429	0.23069
Aminoacyl-tRNA biosynthesis	48	5	0.002199	2.6579	0.026382	0.01429	0.16667
Valine, leucine and isoleucine biosynthesis	8	2	0.004022	2.3956	0.044238	0.017427	0
Primary bile acid biosynthesis	46	1	0.020969	1.6784	0.20969	0.068148	0
Histidine metabolism	16	1	0.038361	1.4161	0.34525	0.083116	0.22131
beta-Alanine metabolism	21	1	0.038361	1.4161	0.34525	0.083116	0
Biotin metabolism	10	1	0.11743	0.93022	0.82202	0.18591	0
Valine, leucine and isoleucine degradation	40	1	0.12871	0.8904	0.82202	0.18591	0
Pantothenate and CoA biosynthesis	19	1	0.12871	0.8904	0.82202	0.18591	0
Lysine degradation	25	2	0.16394	0.78532	0.82202	0.19433	0
Glyoxylate and dicarboxylate metabolism	32	1	0.19433	0.71147	0.82202	0.19433	0.04233

Cysteine and methionine metabolism	33	1	0.19433	0.71147	0.82202	0.19433	0.02184
Sphingolipid metabolism	21	1	0.19433	0.71147	0.82202	0.19433	0

HFHS vs control diet in sham rats							
	Total Compound[1]	Hits[2]	Raw p[3]	-Log (p)	Holm adjust	FDR	Impact[4]
Synthesis and degradation of ketone bodies	5	1	0.000273	3.564	0.002183	0.001092	0
Butanoate metabolism	15	1	0.000273	3.564	0.002183	0.001092	0
Tryptophan metabolism	41	1	0.004535	2.3434	0.027211	0.012094	0.14305
Primary bile acid biosynthesis	46	1	0.007022	2.1535	0.035111	0.014044	0
Aminoacyl-tRNA biosynthesis	48	2	0.032637	1.4863	0.13055	0.05222	0
Valine, leucine and isoleucine degradation	40	1	0.83955	0.075951	1	0.83955	0
Valine, leucine and isoleucine biosynthesis	8	1	0.83955	0.075951	1	0.83955	0
Pantothenate and CoA biosynthesis	19	1	0.83955	0.075951	1	0.83955	0

HFHS+ GSM vs HFHS diet in sham rats							
	Total Compound[1]	Hits[2]	Raw p[3]	-Log (p)	Holm adjust	FDR	Impact[4]
Valine, leucine and isoleucine degradation	40	1	0.056828	1.2454	0.45462	0.15154	0
Valine, leucine and isoleucine biosynthesis	8	1	0.056828	1.2454	0.45462	0.15154	0
Pantothenate and CoA biosynthesis	19	1	0.056828	1.2454	0.45462	0.15154	0
Aminoacyl-tRNA biosynthesis	48	2	0.088865	1.0513	0.45462	0.17773	0
Tryptophan metabolism	41	1	0.26192	0.58183	1	0.41907	0.14305
Primary bile acid biosynthesis	46	1	0.39651	0.40174	1	0.52868	0
Synthesis and degradation of ketone bodies	5	1	0.64199	0.19247	1	0.64199	0
Butanoate metabolism	15	1	0.64199	0.19247	1	0.64199	0

Control+ GSM vs Control diet in sham rats.							
	Total Compound[1]	Hits[2]	Raw p[3]	-Log (p)	Holm adjust	FDR	Impact[4]
Valine, leucine and isoleucine degradation	40	1	0.000159	3.7997	0.001269	0.000423	0
Valine, leucine and isoleucine biosynthesis	8	1	0.000159	3.7997	0.001269	0.000423	0
Pantothenate and CoA biosynthesis	19	1	0.000159	3.7997	0.001269	0.000423	0
Aminoacyl-tRNA biosynthesis	48	2	0.002781	2.5558	0.013904	0.005562	0
Primary bile acid biosynthesis	46	1	0.082918	1.0814	0.33167	0.13267	0
Tryptophan metabolism	41	1	0.13277	0.87691	0.3983	0.17702	0.14305
Synthesis and degradation of ketone bodies	5	1	0.2773	0.55705	0.5546	0.2773	0
Butanoate metabolism	15	1	0.2773	0.55705	0.5546	0.2773	0

[1]Total compound is the number of compounds involved in the pathway.
[2]Hits is the matched number from the user uploaded data.
[3]The raw p is the original p-value calculated from the enrichment analysis.
[4]Impact value is calculated from pathway topology analysis for comparison among
different pathways. It represents the cumulative percentage of importance for the matched metabolite nodes involved in a pathway. The importance of each metabolite node is calculated from centrality measures and represents the percentage with regard to the total pathway importance.
