# Supplementary figures and images for: Mass Spectrometry-Based Metabolomic and Lipidomic Analysis of the Effect of High Fat/High Sugar Diet and GreenshellTM Mussel Feeding on Plasma of Ovariectomized Rats
Source: Metabolites. 2021 Oct 31;11(11):754. doi: 10.3390/metabo11110754 (PMC8622240; doi:10.3390/metabo11110754)

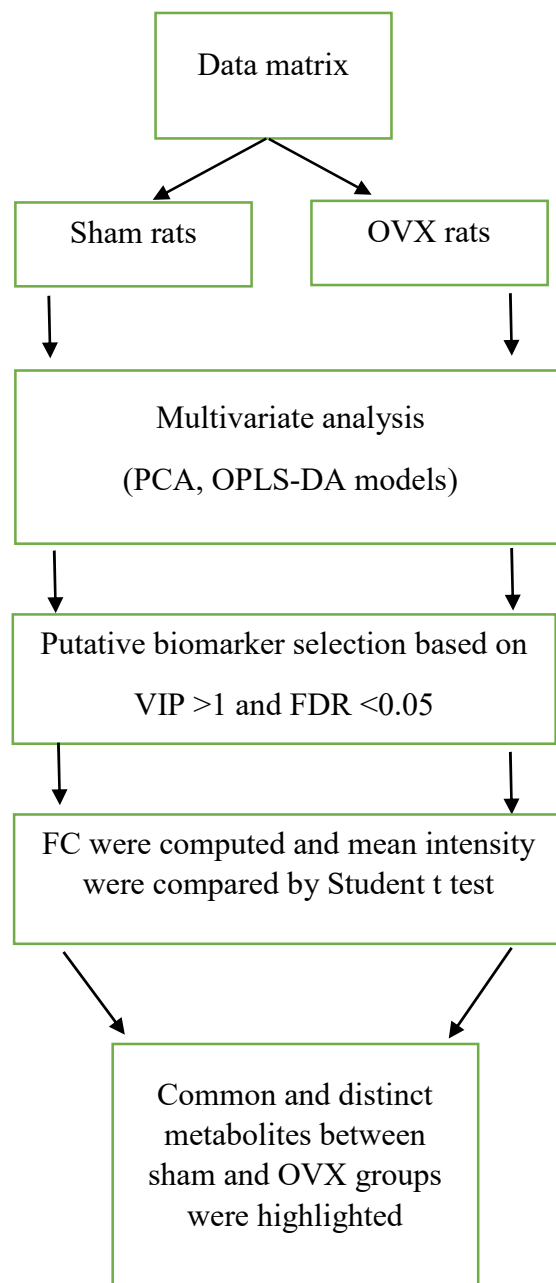

Figure S3. Flowchart of data analysis in metabolomics.

Supplement: Supplementary file 1 [file metabolites-11-00754-s001.zip › Figure S3.pdf]
